# It is Time for a Universal Nutrition Policy in Very Preterm Neonates during the Neonatal Period? Comment on: “Applying Methods for Postnatal Growth Assessment in the Clinical Setting: Evaluation in a Longitudinal Cohort of Very Preterm Infants” *Nutrients* 2019, *11*, 2772

**DOI:** 10.3390/nu12040980

**Published:** 2020-04-02

**Authors:** Antonios Gounaris, Rozeta Sokou, Polytimi Panagiotounakou, Ioanna N. Grivea

**Affiliations:** 1 Neonatal Clinic-NICU, University Hospital of Larissa, 413 34 Larissa, Greece; ioanna.grivea@gmail.com; 2 Neonatal Clinic-NICU, Nikaia General Hospital “Agios Panteleimon”, 184 54 Piraeus, Greece; sokourozeta@yahoo.gr (R.S.); ppppolytimi04@gmail.com (P.P.)

We have read the article entitled “Applying Methods for Postnatal Growth Assessment in the Clinical Setting: Evaluation in a Longitudinal Cohort of Very Preterm infants” by Montserrat Izquierdo Renau et al. published in *Nutrients* [1].

It is an interesting paper which surprised us with the high prevalence of extrauterine growth restriction (EUGR) in the whole sample, which reaches 56.5% at discharge. Unfortunately, the authors have not provided reliable answers for the causes of this high incidence and furthermore offer no suggestions on how to decrease it, despite the fact that EUGR is firmly correlated at least with negative neurodevelopmental outcome [2].

The authors described as potential predictors of EUGR, the gestational age and the need of oxygen supply during admission, factors that are nonmodifiable. As far as the lower provision of lipids during the first week of life is concerned, the quantity suggested in the paper of 3–3.5 g/kg/day is acceptable worldwide. The same question arises from the next correlation of EUGR in the paper with the maximum percentage of initial weight loss. In this six-year study period (2011–2016), the absence of a realistic suggestion for reducing the high percentage of EUGR creates the impression that EUGR is the predetermined outcome for very preterm neonates (VPN).

We have objections to this and for several policies suggested in this paper as we believe that these may be responsible for this high EUGR.

Our main objection is the lower amount of milk administered during the full enteral feeding period (160–180 mL/kg/day). According to both the WHO (in 2013) [3] and recently from Professor Neena Modi [4], at least 200 mg/kg/day of maternal milk should be offered when VPN reach full enteral feeding. In a recent publication by our team, in some hospitalized VPN who did not have the expected growth, the quantity of milk was increased to more than 200 mg/kg/day [5]. This “aggressive” nutrition that was implemented during the 40–44 weeks postconceptional age (PCA) with the persistent nasal continuous positive airway pressure (nCPAP) use in unstable VPN contributed to EUGR (<10th centile) 25% for the body weight and 4.6% for the head circumference at discharge [5].

Fluid policy implemented in the paper could have been responsible for the high percentage of initial weight loss, without improving the prevalence of patent ductus arteriosus (more than 40%) of neonates with birth weight (BW) < 1500 g. A decrease of fluids (145–155 mL/kg/day) for the PDA treatment further influenced growth. Hansson et al. reported that fluid restriction for PDA treatment affected negatively energy intakes and growth in VPN [6].

The administration of donor milk for twenty-eight days in neonates with BW < 1000 g and specifically without fortifier during the first two weeks of life is crucial, as the quantity of protein in donor milk is significantly lower than that in mother’s own milk [7]. In a very recent paper, Li et al. found that VPN with breast feeding exclusively had significantly less body weight at discharge, comparing to predominantly formula-fed neonates without any difference in adipose tissue mass. Authors concluded that the slower weight gain at discharge of VPN fed with breast milk appears to be due to a deficit in nonadipose tissue mass and may reflect the lower protein intake [8].

In conclusion, the high prevalence of EUGR in this paper is perhaps due to both the quantity and quality of the milk administered. Growth of VPN must be closely followed during the 40 weeks PCA and the content of milk (calories, protein) given should be individualized aiming to minimize the percentage of infants with EUGR.
